# Coordination of fungal biofilm development by extracellular vesicle cargo

**DOI:** 10.1038/s41467-021-26525-z

**Published:** 2021-10-29

**Authors:** Robert Zarnowski, Andrea Noll, Marc G. Chevrette, Hiram Sanchez, Ryley Jones, Hanna Anhalt, Jen Fossen, Anna Jaromin, Cameron Currie, Jeniel E. Nett, Aaron Mitchell, David R. Andes

**Affiliations:** 1 Department of Medicine, Section of Infectious Diseases, University of Wisconsin–Madison, Madison, WI USA; 2 Department of Medical Microbiology and Immunology, University of Wisconsin–Madison, Madison, WI USA; 3 Wisconsin Institute for Discovery and Department of Plant Pathology, University of Wisconsin–Madison, Madison, WI USA; 4 Department of Lipids and Liposomes, Faculty of Biotechnology, University of Wroclaw, Wroclaw, Poland; 5 Department of Bacteriology, University of Wisconsin, Madison, WI USA; 6 Department of Biology, University of Georgia, Athens, GA USA

**Keywords:** Fungal biology, Biofilms, Cellular microbiology, ESCRT

## Abstract

The fungal pathogen *Candida albicans* can form biofilms that protect it from drugs and the immune system. The biofilm cells release extracellular vesicles (EVs) that promote extracellular matrix formation and resistance to antifungal drugs. Here, we define functions for numerous EV cargo proteins in biofilm matrix assembly and drug resistance, as well as in fungal cell adhesion and dissemination. We use a machine-learning analysis of cargo proteomic data from mutants with EV production defects to identify 63 candidate gene products for which we construct mutant and complemented strains for study. Among these, 17 mutants display reduced biofilm matrix accumulation and antifungal drug resistance. An additional subset of 8 cargo mutants exhibit defects in adhesion and/or dispersion. Representative cargo proteins are shown to function as EV cargo through the ability of exogenous wild-type EVs to complement mutant phenotypic defects. Most functionally assigned cargo proteins have roles in two or more of the biofilm phases. Our results support that EVs provide community coordination throughout biofilm development in *C. albicans*.

## Abstract (web summary)

The fungal pathogen *Candida albicans* can release extracellular vesicles that promote biofilm formation and antifungal resistance. Here, Zarnowski et al. define functions for numerous vesicle cargo proteins in biofilm matrix assembly and drug resistance, as well as in fungal cell adhesion and dissemination.

## Introduction

Extracellular vesicles (EVs) mediate non-conventional transport of biologically active molecules that are contained in small lipid bilayers following their release from cells. EVs are found across all domains of life, and most microbes produce EVs as an integral part of their lifecycle^1–4^. EVs are known to play diverse roles in delivery of effectors to target cells^5–7^, but the complexity of cargo composition limits the feasibility of analyzing the function of individual components. As a result, knowledge of the roles of specific EV content remains fragmentary.

The predominant growth environment for most microbes is in the setting of surface-associated biofilm communities^8^. These cell populations are commonly high-density and characterized by the encasement of organisms within a polymeric matrix. The microbially-produced matrix frequently affords protection from antimicrobial therapy and other external stressors. In the medical setting, biofilm formation and the associated drug resistance results in disease persistence. Our work focuses on *Candida*, the most common pathogenic fungal biofilm pathogen encountered in patients^9–11^. *Candida* biofilm defies treatment, a property linked to the protective extracellular matrix, with high mortality in patients with retained biofilms^12^. In addition to matrix production and propagation of surface-associated communities, the dynamic process of biofilm formation also involves community dissipation. During disease, cells dispersed from device-associated biofilms serve as the nidus for disseminated, invasive disease. Previous work found EV content of *C. albicans* differs substantially as the organism transitions from the planktonic to biofilm growth^13^. Subsequent investigation demonstrated *Candida* biofilm EVs to promote extracellular matrix formation and associated resistance to antifungal drugs. For the current work, we examined the influence of EVs throughout the entire natural course of biofilm infection.

In this work, we define individual EV cargo proteins responsible for matrix assembly and biofilm-associated drug resistance. We also discover roles for cargo proteins in adhesion and dissemination. Our results indicate that the ESCRT pathway selects EV cargo that coordinates community activity throughout the process of biofilm development.

## Results

### ESCRT pathway directs vesicle cargo composition

**Fig. 1:**
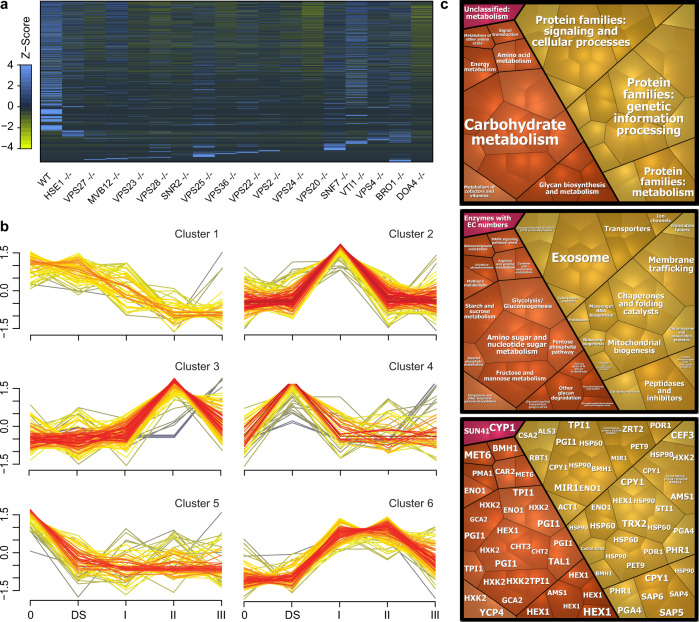
ESCRT-driven extracellular vesicle cargo proteins for genotypic/phenotypic evaluation. **a** A heatmap displaying differential expression of 904 proteins found in the EV proteomes of *C*. *albicans* wild-type and ESCRT mutants. Columns are colored according to the relative protein expression level of each protein. Blue and yellow indicate higher and lower expression Z-scores, and black indicates missing proteins. The color intensity indicates the degree of protein up- or downregulation. Proteomic data were mapped using Heatmapper. **b** The Mfuzz-based grouping of 904 profiles of proteins identified in the EV proteomes of *C*. *albicans* wild-type and ESCRT mutant biofilms. Soft clustering yielded six cluster bins, which consisted of 7–22 protein targets with abundance levels enriched in individual ESCRT complexes. Each line represents an individual protein, where the x-axis is the stage assayed and the y-axis is a z-score of the normalized protein abundance in the proteome. Six clusters of proteins that behave similarly across conditions are shown, with lines colored depending on how a protein fits the cluster average (red = strong fit, gray = weak fit). For example, proteins in Cluster 2 show baseline protein abundance in stages 0, DS, II, and III and increased protein abundance in stage I. Given that the protein cargo profile in extracellular vesicles changes during processing in the ESCRT system, we asked if the effect of individual ESCRT subclasses in extracellular vesicle cargo could be differentiated based on the abundance levels of proteins. In order to bin proteins into groups, we implemented fuzzy clustering based on their abundances across all ESCRT mutants and wild type. Fuzzy clustering yielded a set of 61 protein candidates with abundance levels strongly dependent on individual ESCRT subsystems. **c** KEGG-based functional characterization of 61 select EV cargo protein candidates. Voronoi treemap layouts show smallest clusters that represent the select EV cargo proteins and are arranged inside higher-level regions according to their KEGG functional categories and assignments. Proteins grouped based on hierarchical systems of KEGG PATHWAY are shown in orange and those representing KEGG BRITE are shown in yellow. Proteins listed in KEGG databases without assigned known metabolic function were also included (in pink).

*C. albicans* release distinct extracellular v esicles during biofilm growth, which deliver an adhesive extracellular matrix that renders the encased cells resistant to antifungal therapy^13^. This community phenomenon is genetically dependent on the endosomal sorting complexes required for transport (ESCRT) pathway^14^. To identify EV cargo that may be involved in this process, we performed proteomic analysis of biofilm EVs, comparing wild-type *C. albicans* to 17 different ESCRT mutants. On a per vesicle basis, we found marked variation in their relative protein abundance (Fig. 1a and Supplementary Data 1). On average, more than 60% of the vesicle cargo proteome present in wild-type vesicles was absent in ESCRT mutants (ranging 35–89% among the individual ESCRT mutants) (Supplementary Data 2). In addition, 40% of the proteins that were present in both wild-type and ESCRT mutant EVs showed quantitative differences in abundance. The results implicate the ESCRT pathway in selection of biofilm EV cargo in addition to vesicle transport.

Because ESCRT mutants exhibit biofilm alterations^13^, differentially abundant groups of proteins in their EVs may influence key biofilm processes. We utilized an unsupervised machine-learning algorithm, termed fuzzy clustering, to identify patterns of protein abundance among the biofilm EVs (Supplementary Data 3). The ESCRT mutants group into categories (0, DS, I, II, III) based on their detailed roles in vesicle transport^15^. We included this grouping system in our analysis. Among the more than 900 vesicle proteins, clustering analysis revealed six bins consisting of 7–22 proteins (Fig. 1b). These 63 differentially abundant proteins had a broad range of functional annotations, from carbohydrate modification to membrane trafficking (Fig. 1c and Supplementary Data 4). Notably, some cargo clusters were enriched in EVs of ESCRT mutant classes (e.g., enrichment of cluster 2 cargo in ESCRT-I mutant EVs) and may provide insight into differences among ESCRT mutant phenotypes. Mechanistic connections of most groups of cargo proteins to biofilm development is not obvious, but more than 40% have been linked to biofilm formation in a previous transcript profiling analysis (Supplementary Data 3)^16^. These observations suggest that EV cargo is adapted to community needs.

### EV cargo proteins impact biofilm extracellular matrix production and drug resistance

**Fig. 2:**
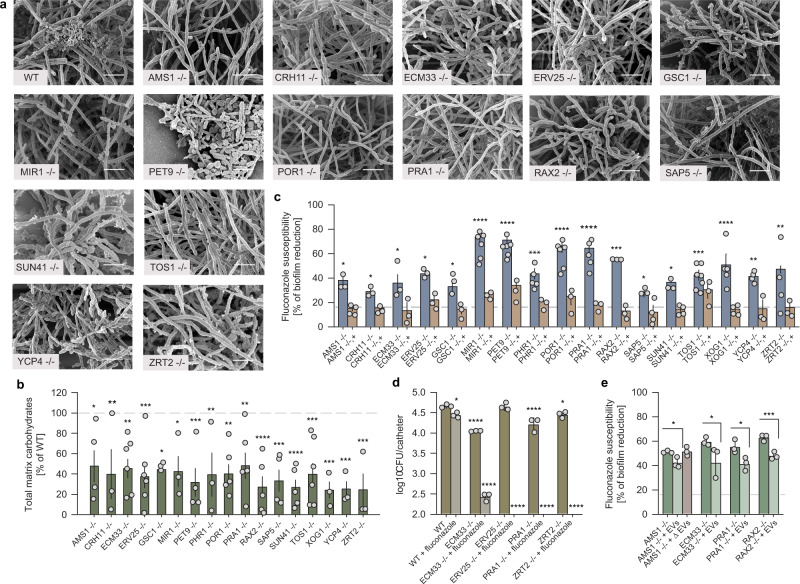
Impact of extracellular vesicle protein cargo on biofilm matrix quantity and function. **a** Biofilm matrix surrounding *C*. *albicans* biofilm cells from WT and EV cargo mutant 24-h-old biofilms on in vitro coverslips visualized by SEM. Scale bar = 5 µm. **b** EV cargo proteins contribute to ECM total carbohydrate profiles. The percentage changes in total carbohydrates in biofilm ECM of all EV cargo mutants determined by gas chromatography. Each dot is an independent biological replicate and represents the mean of 2 technical replicates, from left to right *n* = 4, 3, 6, 6, 3, 3, 4, 3, 5, 5, 5, 4, 5, 6, 3, 3, 3. Error bars represent standard deviation. Non-parametric Kruskal–Wallis one-way analysis of variance with uncorrected Dunn’s multiple comparison test was performed. Indicated *p* values, from left to right: 0.0130, 0.0082, 0.0019, 0.0003, 0.0187, 0.0132, 0.0004, 0.0075, 0.0011, 0.0068, <0.0001, 0.0006, <0.0001, 0.0005, 0.0004, 0.0005, and 0.0004. **c** The percent of reduction in biofilm formation following treatment with 1000 μl/ml fluconazole compared with untreated biofilms. The null deletions and corresponding complemented strains are shown for mutants with enhanced susceptibility phenotype. Each dot is an independent biological replicate and represents the mean of 3 technical replicates, from left to right *n* = 3, 4, 3, 3, 3, 3, 3, 3, 3, 3, 6, 3, 6, 3, 5, 3, 6, 3, 5, 3, 3, 3, 3, 4, 3, 3, 7, 3, 5, 4, 4, 3, 4, 3. Error bars represent standard deviation. Non-parametric Kruskal–Wallis one-way analysis of variance with uncorrected Dunn’s multiple comparison test was performed. Indicated *p* values, from left to right: 0.0179, 0.0145, 0.0131, 0.0104, 0.0203, <0.0001, <0.0001, 0.0010, <0.0001, <0.0001, <0.0001, 0.0240, 0.0420, 0.0004, <0.0001, 0.0079, and 0.0022. **d** Quantification of in vivo biofilms following antifungal therapy using a rat venous catheter model. Select fluconazole-susceptible EV protein cargo mutants were treated either with fluconazole 250 μg/ml or 0.9 M NaCl followed by the CFU analysis. Three animals and culture replicates per condition, *n* = 3. Error bars represent standard deviation. Non-parametric Kruskal–Wallis one-way analysis of variance with uncorrected Dunn’s multiple comparison test was performed. Indicated *p* values, from left to right: 0.0122, <0.0001, <0.0001, <0.0001, <0.0001, <0.0001, 0.0219, and <0.0001. **e** Effect of exogenous WT biofilm EVs on biofilm fluconazole susceptibility for select ESCRT null mutants as measured by the 96-well XTT assay. Biofilm cultures of fluconazole-sensitive mutant strains amended with WT EVs regain their ability to grow in the presence of fluconazole. Each dot is an independent biological replicate and represents the mean of 3 technical replicates, *n* = 3. Error bars represent standard deviation. Unpaired two-tailed *t*-test and non-parametric Kruskal–Wallis one-way analysis of variance with uncorrected Dunn’s multiple comparison test were performed. Indicated *p* values, from left to right: 0.0202, 0.0174, 0.0351, and 0.0032.

We sought to test our hypothesis that select biofilm EV cargo function in biofilm matrix biogenesis and associated antifungal drug resistance^17^. We constructed homozygous deletion mutants for the 63 proteins identified in our ESCRT cargo clustering analysis and analyzed their biofilm phenotypes. Mutants of cargo protein genes *GSC1* (*FKS1*), *PHR1, SUN41*, and *XOG1* were included as internal controls; we had shown previously that these polysaccharide biogenesis proteins are necessary for matrix production and protection from antifungals^13,18^. We assessed all mutants’ biofilm formation capacity, biofilm drug susceptibility to the antifungal fluconazole, and assembly of matrix polysaccharides (Fig. 2a–c). All mutants formed biofilms, and the biofilm biomass of most mutants was comparable to that of the wild type (Supplementary Data 5). However, biofilms of 13 new cargo mutants showed increased drug susceptibility (Fig. 2c). Their increased drug susceptibility was reversed by genetic complementation (Fig. 2c), thus validating the mutants’ genotype–phenotype relationship. Scanning electron microscopic imaging of these mutant biofilms suggested that they had reduced content of extracellular matrix material (Fig. 2a). Matrix quantification confirmed that all mutants with drug hypersusceptible biofilms had significantly diminished major polysaccharide (Fig. 2b and Supplementary Fig. 1). Vesicle quantity was relatively similar or higher among mutants compared to the reference strain, suggesting the representative cargo and not simply vesicle number was responsible for the phenotype (Supplementary Fig. 2). Planktonic minimum inhibitory concentrations (MIC) were unchanged for this group of cargo mutants (Supplementary Data 6)^17^. These data indicate that 17 of the 63 cargo protein genes, are required for biofilm matrix accumulation and biofilm-specific drug resistance.

We assessed the clinical relevance of our findings in a rodent venous catheter infection model that mimics one of the most common biofilm infections in patients^19^. We tested mutants of four representative cargo protein genes: *ECM33*, *ERV25, PRA1*, and *ZRT2*. These mutants produced biofilms in the in vivo model with burdens close to wild type (Fig. 2d). However, treatment with fluconazole reduced mutant biofilms by 30-fold or more, whereas wild-type biofilms were minimally impacted. Therefore, cargo protein mutant biofilm defects are consistent in both in vitro and in vivo settings.

Our hypothesis is that EV cargo proteins participate in matrix production through their presence in EVs. An alternative possibility is that EV cargo proteins function in matrix production through their established cellular localization; their presence in EVs may be unrelated to matrix production. To test our hypothesis, we utilized vesicle “add-back” assays to determine whether addition of wild-type biofilm EVs rescues cargo mutant drug sensitivity^13^. Assay sensitivity was maximized by choosing mutants with the strongest phenotype. Addition of wild-type vesicles restored significant biofilm resistance for the *ams1*Δ/Δ, *ecm33*Δ/Δ, *pra1*Δ/Δ, and *rax2*Δ/Δ mutant biofilms (Fig. 2e). Conversely, exogenous administration of mutant EVs to the mutant biofilm (ams1Δ/Δ EVs) did not alter the biofilm drug susceptibility (Supplementary Fig. 3). Effects were dose-dependent (Supplementary Fig. 4). EV addition did not completely reverse drug sensitivity, an indication that the gene products may also function independently of EV transport, or that EV addition may not fully recapitulate biofilm community physiology. However, these results show that biofilm EVs deliver functional cargo to promote biofilm drug resistance.

### Coordination of biofilm development by EVs and their cargo

Biofilm community formation occurs in steps that are coordinated through genetic and physiological regulatory mechanisms. We sought to determine whether EVs and their cargo may have roles in community coordination beyond matrix biogenesis. We first assayed a panel of EV-deficient ESCRT mutants for an early event in biofilm development, cell-surface adhesion, and a late event, cell dispersion^20–22^. All ESCRT mutants tested had increased adhesion compared to the wild type, and genetic complementation reduced their adhesion significantly (Supplementary Fig. 5a). In addition, all ESCRT class I and II mutants had reduced dispersion, while most other ESCRT mutants had either reduced or increased dispersion, with phenotypic reversal after genetic complementation (Supplementary Fig. 5b). These results show that the ESCRT complex governs biofilm formation more broadly than previously thought. It has a clear negative role in adhesion, and a mixed role in dispersion.

**Fig. 3:**
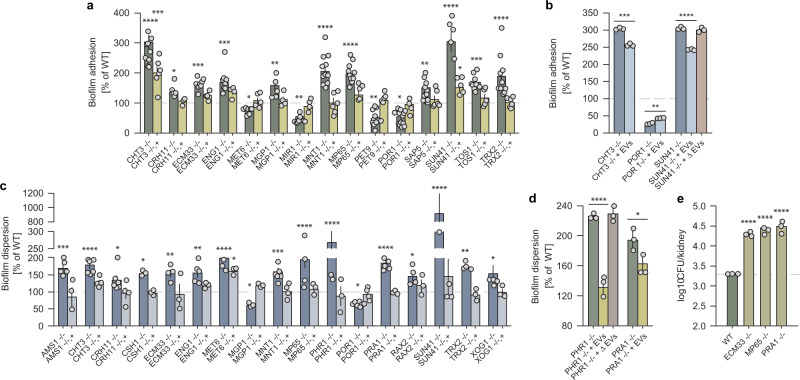
Select extracellular vesicle cargo proteins affect cell adhesion and dispersion from biofilms. **a** The percent of biofilm adhesion over 90 min of incubation of the EV cargo mutants as compared to WT control. Both the null deletion mutants and the corresponding complemented strains are shown. Each dot is an independent biological replicate and represents the mean of 8 technical replicates, from left to right *n* = 11, 6, 7, 6, 12, 5, 10, 6, 9, 5, 5, 5, 14, 5, 12, 6, 11, 3, 12, 6, 13, 5, 8, 6, 6, 4, 9, 5, 10, 6. Error bars represent standard deviation. Non-parametric Kruskal–Wallis one-way analysis of variance with uncorrected Dunn’s multiple comparison test was performed. Indicated *p* values, from left to right: <0.0001, 0.0002, 0.0180, 0.0001, 0.0002, 0.0483, 0.0032, 0.0065, <0.0001, <0.0001, 0.0075, 0.0119, 0.0062, <0.0001, 0.0380, 0.0003, and <0.0001. **b** Effect of exogenous WT biofilm EVs on biofilm adhesion for select ESCRT null mutants as measured by the 96-well XTT assay. Biofilm cultures of select mutant strains with altered adhesion amended with WT EVs return toward WT adhesion capacity. Each dot is an independent biological replicate and represents the mean of 8 technical replicates, *n* = 3. Error bars represent standard deviation. Unpaired two-tailed *t*-test was performed. Indicated *p* values, from left to right: 0.0001, 0.0085, and <0.0001. **c** The percent of fungal cell dispersion from mature biofilms at 24 h. The ESCRT mutants are shown as compared to WT control. Both the null deletion mutants and the corresponding complemented strains are shown. Each dot is an independent biological replicate and represents the mean of 8 technical replicates, from left to right *n* = 5, 3, 7, 4, 6, 4, 3, 3, 4, 3, 5, 3, 6, 3, 3, 3, 7, 4, 7, 3, 5, 3, 7, 4, 7, 3, 4, 3, 5, 4, 4, 3, 5, 3. Error bars represent standard deviation. Non-parametric Kruskal–Wallis one-way analysis of variance with uncorrected Dunn’s multiple comparison test was performed. Indicated *p* values, from left to right: 0.0007, <0.0001, 0.0273, 0.0206, 0.0088, 0.0033, <0.0001, 0.0106, 0.0461, 0.0006, <0.0001, <0.0001, 0.0464, <0.0001, 0.0203, <0.0001, 0.0010, and 0.0117. **d** Effect of exogenous WT biofilm EVs on biofilm dispersion for select ESCRT null mutants as measured by the 96-well XTT assay. Biofilm cultures of select mutant strains with altered dispersion amended with WT EVs return toward WT dispersion capacity. Each dot is an independent biological replicate and represents the mean of 8 technical replicates, *n* = 3. Error bars represent standard deviation. Unpaired two-tailed *t*-test was performed. Indicated *p* values are <0.0001 and 0.035. **e** CFU analysis of dispersion from rat venous catheter biofilm to ra*t* kidneys. Select cargo mutants with altered in vitro dispersion were examined. Three animal and culture replicates per condition, *n* = 3. Non-parametric Kruskal–Wallis one-way analysis of variance with uncorrected Dunn’s multiple comparison test was performed. Indicated *p* values are <0.0001.

These findings with ESCRT mutants suggest that biofilm EVs and their cargo may govern adhesion and dispersion. We found that 15 cargo mutants altered adhesion significantly (Fig. 3a); the preponderance of these mutants caused increased adhesion like the ESCRT mutants. Genetic complementation partially or fully reversed the altered adhesion. Addition of wild-type EVs partially reversed the adhesion defects of three mutants tested (Fig. 3b); effects were dose-dependent (Supplementary Fig. 4). However, exogenous administration of mutant EVs to the mutant biofilm (sun41Δ/Δ EVs) did not alter biofilm adhesion (Supplementary Fig. 3). These data argue that EVs are at least partially responsible for these cargo mutant adhesion alterations.

We also assayed biofilm dispersion. We found that 17 cargo mutants altered biofilm dispersion significantly, and that genetic complementation reversed the phenotypic alteration (Fig. 3c). Almost all of the mutants displayed increased dispersion. Addition of wild-type EVs substantially reversed the increased dispersion of two representative mutants (Fig. 3d and Supplementary Fig. 4). Exogenous administration of mutant EVs to the mutant biofilm (phr1Δ/Δ EVs) did not, however, alter biofilm dispersion (Supplementary Fig. 3). These data indicate that EVs mediate the functions of cargo proteins in control of biofilm dispersion.

We evaluated the clinical relevance of the cargo mutant dispersion increases with the rat central venous catheter model^19^. Dispersion from biofilm was measured through analysis of fungal burden in the kidney. The model mimics the clinical setting in which patients with biofilm device infections suffer disseminated disease following the dispersion of organisms from biofilm. In vivo testing with three ‘high’ in vitro dispersion mutants (*ecm33*Δ/Δ, *mp65*Δ/Δ, *pra1*Δ/Δ) demonstrated more than a 10-fold increase in kidney burden compared to the wild type (Fig. 3e). These observations support a role for vesicle cargo in dispersion under clinically relevant in vivo conditions.

## Discussion

**Fig. 4:**
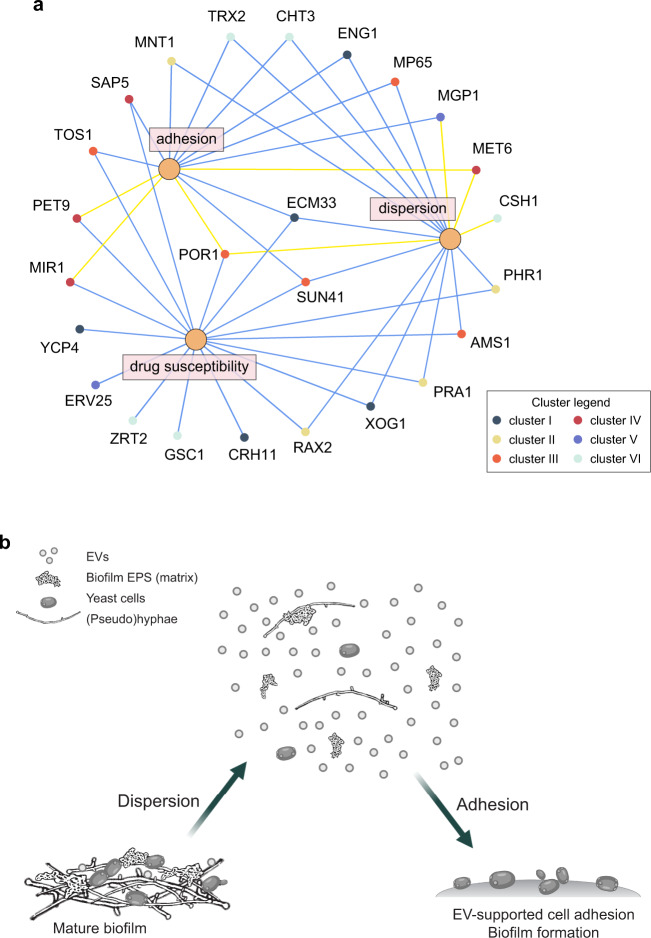
A schematic model summarizing common and unique activities of EV cargo in *C. albicans* biofilm biology. **a** Network diagram illustrating the shared and distinct functions of select EV cargo proteins. Biofilm functional categories considered include adhesion, drug resistance, and cell dispersion. **b** EV biofilm function based on the observation that EVs contain fungal protein cargo constituents that are secreted into the intracellular milieu. EVs are released during biofilm growth and maturation, and alterations of the EV cargo affect the ECM profile, this modulates cell adhesion, matrix delivery, and associated drug resistance as well as cell dissemination from the biofilm. Created with BioRender.com.

Our studies here reveal that EVs have multiple roles in *C. albicans* biofilm development, and that EV functions are mediated by specific cargo proteins. Studies of bacteria have shown previously that EVs have a role in biofilm formation, as have our own previous studies of *C. albicans*^13,23^. However, to our knowledge, no other study has shown that the addition of exogenous vesicles can modulate multiple biofilm-associated attributes, nor has any study assigned vesicle-conveyed functions for multiple individual cargo proteins. It is well appreciated that a transcriptional network governs events in biofilm development; key transcription factors, alone or together, activate target genes required for the yeast-hyphal transition, adherence, drug resistance, and dispersion. Our studies here indicate that there is a parallel EV network that influences many of the same events, that EVs act through “target” cargo proteins, and that by its nature the EV network is able to relay information among members of the biofilm community (Fig. 4).

In our proposed view of an EV regulatory network for biofilm development, the ESCRT machinery may be considered analogous to the transcription factors of the transcriptional network. We have shown previously that, as in other eukaryotes, ESCRT defects lead to EV defects^13^. Our studies here refine that understanding through the finding that individual ESCRT mutants yield residual EVs with distinct cargo. This observation implies that ESCRT subunits or subcomplexes have a role in cargo selection. That role may be direct, through cargo protein binding, as occurs with ESCRT-0 subunits during multivesicular body formation, or indirect, through physiological impact of an ESCRT defect.

The distinctive impact of individual ESCRT defects on cargo selection may explain why biofilm dispersion is increased by some ESCRT mutations and decreased by others. Although our functional map of cargo proteins is far from complete, our data provide an encouraging suggestion that ESCRT mutant phenotypes correlate with cargo functions. For example, we found that ESCRT-I defects cause decreased dispersion, and that cargo defects *phr1*Δ/Δ and *pra1*Δ/Δ cause increased dispersion. Hence Phr1 and Pra1 are inhibitors of dispersion. Phr1 and Pra1 are in cargo cluster 2, which is enriched in ESCRT-I mutants but not in other ESCRT mutants. Similarly, ESCRT-II defects cause decreased dispersion, and those EVs are enriched in Sun41, another inhibitor of dispersion. A simple model is that ESCRT-I and ESCRT-II defects cause decreased dispersion because they are enriched in these inhibitors of dispersion.

EVs are well suited to coordinate biofilm developmental regulation. One key feature is that their concentration provides an indication of local cell density, and consequently allows cells to make an appraisal of biofilm formation feasibility. A second feature is that biofilm-specific EV content can indicate whether a biofilm has already formed nearby, informing a possible cellular decision between joining an existing biofilm or creating a new one. Thus, in addition to mediating resource sharing for matrix biogenesis^13^, our results here suggest that EVs have informational roles that broadly shape community-based physiological behavior. This view of EVs and their cargo as regulators of functions of diverse biofilm-associated events may point to new therapeutic avenues that target multiple phases of biofilm growth.

## Methods

### Media and culture conditions

Fungal strains were stored in 15% glycerol frozen at −80 °C. Strains were routinely maintained on YPD agar plates (1% yeast extract, 2% Bacto™ peptone, 2% dextrose, 2% Bacto™ agar), whereas liquid cultures were grown in broth YPD (1% yeast extract, 2% Bacto™ peptone, 2% dextrose) rotating at 200 rpm at 30 °C. For biofilm assays, strains were cultured in filter sterilized Roswell Park Memorial Institute medium 1640 (RPMI), buffered with 4-morpholinepropanesulfonic acid (MOPS), and pH adjusted to 7.0^24^.

### Fungal strains and mutant construction

All fungal strains used in this study are listed in Supplementary Data S7. The *C. albicans* background strain SN152 (MTL a/α) was used to create homozygous deletion strains using a splicing by overhang extension (SOE)-PCR-based disruption method with the pSN52 *C. dubliniensis* HIS1 and pSN40 *C. maltosa* LEU2 auxotrophic markers^25^. Complementation of mutant strains with a single allelle of a gene-of-interest was achieved using the pSN69 *C. dubliniensis* ARG4 auxotrophic marker. SOE-PCR-amplified cassettes were transformed into *C. albicans* cells using the lithium acetate transformation method^26^ and transformants obtained were selected on respective minimal media (0.7% yeast nitrogen base without amino acids, 0.2% amino acid stock mix, 2% dextrose, 2% Bacto™ agar) amended with the required auxotrophic supplements, if needed. PCR with primers listed in Supplementary Data 8 was used to verify genotypes.

### In vitro biofilm models

Biofilms were grown in one of four models: 96-well or 6-well polystyrene plate, polystyrene roller-bottle, or glass coverslip. Ninety-six-well flat-bottom polystyrene plates were used to assess biofilm treatment effects^27,28^. The 6-well plate assay was used to assess matrix composition. The coverslip assay was used for in vitro biofilm SEM imaging. A rolling bottle system was used to generate matrix for analyses^24^. Briefly, aliquots of *C. albicans* grown in RPMI-MOPS were used to inoculate a polystyrene roller. Bottles were placed on a roller apparatus (Wheaton Science Products, Millville, NJ), rolling at the rate of 20 rpm at 37 °C. After 24 h, the biofilm culture medium was replaced with fresh media and the bottles were incubated for another 24 h. At least three biological and technical replicates were performed for each assay.

### Label-free gel-free proteomics

Following matrix and vesicle isolation, enzymatic “in liquid” digestion and mass spectrometric analysis was done at the Mass Spectrometry Facility, Biotechnology Center, University of Wisconsin–Madison. Two hundred micrograms of proteins were extracted by precipitation with 15% Trichloroacetic acid (TCA)/60% acetone and then incubated at −20 °C for 30 min. The matrix or vesicle preparation was centrifuged at 16,000 × *g* for 10 min, and the resulting pellets were washed twice with ice-cold acetone, followed by an ice-cold MeOH wash. Pelleted proteins were resolubilized and denatured in 10 μl of 8 M urea in 100 mM NH_4_HCO_3_ for 10 min, then diluted to 60 μl for tryptic digestion with the following reagents: 3 μl of 25 mM DTT, 4.5 μl of acetonitrile, 36.2 μl of 25 mM NH_4_HCO_3_, 0.3 μl of 1 M Tris-HCl, and 6 μl of 100 ng/μl Trypsin Gold solution in 25 mM NH_4_HCO_3_ (Promega). Digestion was conducted in two stages, first overnight at 37 °C, then additional 4 μl of trypsin solution were added and the mixture was incubated at 42 °C for an additional 2 h. The reaction was terminated by acidification with 2.5% TFA to a final concentration of 0.3% and then centrifuged at 16,000 × *g* for 10 min. Trypsin-generated peptides were analyzed by nanoLC-MS/MS using the Agilent 1100 nanoflow system (Agilent) connected to a hybrid linear ion trap-orbitrap mass spectrometer (LTQ-Orbitrap, Thermo Fisher Scientific) equipped with a nanoelectrospray ion source. Capillary HPLC was performed using an in-house fabricated column with an integrated electrospray emitter, as described elsewhere^29^. Sample loading and desalting were achieved using a trapping column in line with the autosampler (Zorbax 300SB-C18, 5 μm, 5 × 0.3 mm, Agilent). The LTQ-Orbitrap was set to acquire MS/MS spectra in a data-dependent mode as follows: MS survey scans from 300 to 2000 *m*/*z* were collected in profile mode with a resolving power of 100,000. MS/MS spectra were collected on the five most abundant signals in each survey scan. Dynamic exclusion was employed to increase the dynamic range and maximize peptide identifications. Raw MS/MS data were searched against a concatenated *C. albicans* amino acid sequence database using an in-house MASCOT search engine^30^. Identified proteins were further annotated and filtered to 1.5% peptide and 0.1% protein false-discovery-rate with Scaffold Q + version 4.10.0 (Proteome Software Inc.) using the protein prophet algorithm^31^. Proteomic data were mapped using Heatmapper^32^. The mass spectrometry proteomics data have been deposited to the ProteomeXchange Consortium via the partner repository with the dataset identifier PXD028596 and 10.6019/PXD028596^33^.

### Machine learning-based fuzzy clustering

Given that the protein cargo profile in vesicles changes during processing in the ESCRT system, we asked if the effect of individual ESCRT subclasses on vesicle cargo could be differentiated based on the abundance levels of proteins. In order to bin proteins into groups, we implemented fuzzy clustering based on their abundances across all ESCRT mutants and wild type. Soft clustering implemented in R with the Mfuzz package^34^, which performed soft clustering of protein abundance profiles using the fuzzy c-means algorithm based on minimization of a weighted square error function. Data were partitioned into *k*-labeled bins. *k* values from 1 to 20 were evaluated and *k* = 6 was chosen as the point at which explained variance reached diminishing returns (i.e., elbow method). This machine learning strategy identified protein patterns that were correlated with the individual ESCRT subclasses. Overall, fuzzy clustering yielded a set of 63 protein candidates with abundance levels strongly dependent on individual ESCRT subsystems.

### In vitro 96-well microdilution plate-based biofilms and biofilm phenotypic studies

Ninety-six-well flat-bottom polystyrene plates were used to assess biofilm adherence, biofilm dispersion, biofilm susceptibility to drug treatment, and the effect of exogenous vesicles on biofilm drug susceptibility. Fungal cell inocula (10^6^ cells/ml) were prepared out of overnight yeast cultures in YPD at 30 °C, followed by dilution in RPMI-MOPS based on count numbers with an automated Countess™ II cell counter (Invitrogen). One hundred microliters of yeast cells per well were seeded and inoculated plates were incubated depending on their application as follows:

(i) For the adhesion assay, plates were incubated for 90 min at 37 °C^35^. The media was then removed and non-adherent cells were gently washed out with PBS. The density of adhered fungal cells was determined by a tetrazolium salt XTT reduction assay^28,36^. Briefly, solutions of XTT (2,3-Bis[2-methoxy-4-nitro-5-sulfophenyl]-2H-tetrazolium-5-carboxanilide inner salt; 0.75 mg/ml) and PMS (5-methylphenazinium methyl sulfate; 3.20 mg/ml) were prepared fresh for each set of assays and were kept away from light. To each well, 90 μl XTT and 10 μl phenazine methosulfate were added and incubated in the dark at 37 °C for 1 h. Absorbances at 492 nm were measured using an automated Cytation 5 imaging reader (BioTek). Adherence capacity of yeast cells was calculated using the change in absorbance compared to that of controls.
(ii) For the drug susceptibility assay, biofilms were treated with fluconazole, one of the most prescribed antifungal azoles^36^. The drug was used at 1000 µg/ml. After a 6-h biofilm formation period in the wells of 96-well microtiter plates, the biofilms were washed twice with phosphate-buffered saline (PBS, pH 7.2) in order to remove non-adherent cells, followed by the addition of the antifungal drug and fresh RPMI medium. The procedure including the drug treatment was repeated after 24 h and the plates were incubated for an additional period of 24 h, followed by the XTT assay described above. The percent reduction in biofilm growth was calculated using the reduction in absorbance compared to that of controls with no antifungal treatment.
(iii) For the dispersion assay^21^, seeded biofilms were incubated for 6 h at 37 °C, then washed with PBS and fresh RPMI was applied. After continued incubation for another 24 h, the biofilms were washed with PBS and RPMI was replaced with fresh one and allowed to incubate for 24 h at 37 °C. Supernatants were then carefully removed from biofilm cultures and 100-μl aliquots were transferred to a fresh 96-well plate. The amount of dispersed biofilm cells was determined by the modified XTT assay, in which both XTT and PMS were applied at double concentration. Alternatively, dispersion capacity of biofilms was assayed in supernatants collected from 48-h-old non-treated biofilms from the drug susceptibility assay plates. Dispersion capacity of biofilms was calculated using the change in absorbance compared to that of controls.
(iv) For the vesicle addback assay, biofilms were inoculated as described above. After a 5-h biofilm formation period, the biofilms were washed with PBS twice, and purified vesicles diluted in PBS were added. For treatment studies, after an additional hour of incubation, biofilm cultures were treated with fluconazole (1000 μg/ml), followed by the drug treatment protocol described above^13^. Vesicle quantity and size were assessed by imaging flow cytometry, nanosight, and cryo-EM (Supplementary Figure S6). EVs were used at concentrations up to 1,168,088 ± 91,584 particles/ml.

### In vitro 6-well plate-based biofilms and biofilm phenotypic studies

Six-well polystyrene plates were used to assess biofilm extracellular matrix (ECM) carbohydrate composition. Fungal cell inocula were prepared as described above. Mature 48-h-old biofilms were processed depending on their application as follows: Biofilms were seeded with 10^6^ yeast cells per well. The non-adherent cells were removed after a 60-min-long adherence incubation and 1 ml of fresh RPMI medium was applied to each well. The biofilms were grown on an orbital shaker set at 50 rpm at 37 °C for 24 h, then the medium was replaced with fresh RPMI and the incubation was continued for another 24 h. biofilms were removed from wells with a sterile spatula and harvested in sterile water (1 ml/well). The aliquots were combined in a 15-ml Falcon tube and the biofilm biomass was subjected to sonication in a water bath sonicator for 20 min. In order to separate the dissolved ECM from fungal biomass, the sample was centrifuged at 2880 × *g* at 4 °C for 20 min. Five milliliters of the collected ECM suspension was placed in a clear 8-ml glass screw thread vial and dried overnight at 60 °C. Such prepared samples were used for gas chromatography-based carbohydrate profiling as described below.

### Biofilm ECM carbohydrate profiling

Carbohydrates in biofilm ECMs were analyzed based on the modified procedures reported elsewhere^37^. Monosugars were converted to alditol acetate derivatives^38^ and then identified and quantified by gas chromatography on a Shimadzu GC-2010 system (Shimadzu). A Crossbond™ 50% cyanopropylmethyl/50% phenylmethyl polysiloxane column was used (15 m × 0.25 mm with 0.25 μm film thickness, RTX-225, Restek). The GLC conditions were as follows: injector at 220 °C, FID detector at 240 °C, and a temperature program of 215 °C for 2 min, then 4 °C/min up to 230 °C before holding for 11.25 min, run at constant linear velocity of 33.4 cm/s and split ratio of 50:1.

### Biofilm vesicle isolation and characterization

Vesicles were prepared using a large-scale rolling bottle biofilm model system^24^. The culture media was removed from the polystyrene bottles, filter sterilized, and concentrated down to 25 ml using a Vivaflow 200 unit (Sartorius AG) equipped with a Hydrosart 30 kDa cut-off membrane. In order to remove smaller cellular debris, the sample was centrifuged first at 10,000 × *g* for 1 h at 4 °C. The pellet was discarded, and the resulting supernatant was centrifuged again at 100,000 × *g* for 1.5 h at 4 °C. Next, the supernatant was discarded, and the pellet was then resuspended in 10 μl of PBS per bottle. The sample was filter sterilized at stored until further use at 4 °C. Accordingly to the standards for studies of EVs, the isolated EVs were further qualitatively validated by imaging flow cytometry, nanoparticle tracking analysis, and cryo-transmission electron microscopy (Supplementary Fig. 6)^39^. To verify that the observed phenotypic effects in the EV addback assay occurred indeed due to the presence of EVs and was not driven by other co-purified non-vesicular contaminants, the cell-free EV fraction obtained after the ultracentrifugation step (100,000 × *g* for 1.5 h at 4 °C) was further processed using an exoEasy Maxi Kit (Qiagen). This kit uses generic membranes with high affinity for specific binding and recovery of EVs from cell culture supernatant. These EVs were then assessed in the EV addback assay (Supplementary Fig. 6e), which demonstrated comparable activity levels of both membrane-polished EVs and EVs prepared only by ultracentrifugation. Thus, we subsequently removed this step from our EV purification protocol in this study.

The mean particle size of the vesicle dispersions was determined using a Zetasizer Nano-ZS (Malvern Instruments). In order to obtain the optimal light scattering intensity, 10 μl of the vesicle suspension was added to 990 μl of PBS. All the measurements were carried out in triplicate at 25 °C.

Vesicles were quantified using nanoparticle tracking analysis. EV samples were diluted in PBS to a final volume of 1 ml and pretested to obtain an ideal 30–100 particles per frame rate using a NanoSight NS300 system (Malvern). The following settings were applied: camera level was increased to 16 and camera gain to 2 until tested images were optimized and nanoparticles were distinctly visible without exceeding particle signal saturation. Each measurement consisted of five 1-min videos with a delay of 5 s between sample introduction and the start of the first measurement. For detection threshold analysis the counts were limited to 10–100 red crosses and no more than 5–7 blue crosses. Acquired data were analyzed using the NanoSight Software NTA 3.4 Build 3.4.003. At least 1000 events in total was tracked per sample in order to minimize data skewing based on single large particles^40^.

Imaging flow cytometry was used to quantify and assess the overall quality of extracellular vesicles. Prior to analysis, samples were stained with carboxyfluorescein succinimidyl ester (CSFE) and 1,1′-dioctadecyl–3,3,3′,3′-tetramethylindocarbocyanine perchlorate (Dil) at 37 °C for 3 h. Excessive dye particles were removed from stained vesicles using illustra microspin G-50 columns (GE Healthcare). All samples were analyzed on the ImageStreamX Mk II flow cytometry system (Amnis Corporation) at ×60 magnification, with default low flow rate/high sensitivity using the INSPIRE software (Supplementary Fig. 6)^39^.

### Biofilm matrix isolation

Biofilm matrices were used for biochemical analyses were grown in the large-scale rolling bottle model system^24^. After 48 h of growth and media removal, the biofilms were dislodged from the roller-bottle surface with a sterile spatula. The intact biofilms were then gently subjected to sonication in order to remove matrix from fungal cells. Sonication with done with a 6-mm microtip head at 20 kHz with an amplitude of 30% for 8 min, followed by centrifugation to separate the biomass from the matrix, and the isolated matrix was then lyophilized. Matrices collected from 6-well biofilm plates were processed in a similar way^41^.

### Biofilm imaging

The coverslip assay was used for in vitro biofilm imaging^17^. Briefly, in vitro biofilms were grown on sterile coverslips (Thermanox) in 12-well polystyrene plates that pre-coated with 10 µl of human NaEDTA plasma each and allowed to dry at 30 °C. Forty microliters of yeast in RPMI was counted and diluted as in the biofilm models described above and added to each coverslip at 30 °C for 60 min. The initial inoculum was then removed, 1 ml of fresh RPMI containing 5% NaEDTA human plasma was added to each well, and the plates were incubated at 37 °C for 20 h and 50 rpm on an orbital shaker for an additional period of 24 h. A rolling bottle system was used to generate matrix for analyses.

For SEM of biofilms^17^, 40 μl of an inoculum of 10^8^ cells/ml in RPMI was added to the coverslips and incubated at 37 °C for 60 min. One ml RPMI was added to each well, and the plates were incubated at 37 °C for 20 h. One milliliter fixative (4% formaldehyde, 1% glutaraldehyde in PBS) was then added to each well prior to incubation at 4 °C overnight. Coverslips were then washed with PBS prior to incubation in 1% OsO_4_ for 30 min. Samples were then serially dehydrated in ethanol (30–100%). Critical point drying was used to completely dehydrate the samples prior to palladium-gold coating. Samples were imaged on a SEM LEO 1530, with Adobe Photoshop CC (20.0.4 release) used for image compilation.

### EV addback assay

Biofilms were formed in the wells of 96-well microtiter plates, as described above. After a 5 h biofilm formation period, the biofilms were washed with phosphate-buffered saline (PBS) twice and purified EVs at concentrations of 21,804 ± 1711 EVs/ml were added. For treatment studies, after an additional hour if incubation, biofilm cultures were amended with fluconazole (1000 µg/ml) followed by the drug treatment protocol described above. For biofilm matrix studies, the samples were incubated for an additional 24 h prior to either SEM imaging or matrix isolation for quantitative carbohydrate analysis.

### In vivo *Candida* vascular catheter biofilm and kidney dispersion model

In vivo biofilm testing was performed with a rat external jugular venous catheter model^19^. Briefly, a 10^6^ cells/ml inoculum for each strain or strain combination was allowed to grow on an internal jugular catheter placed in a pathogen-free female rat (16-week old, 400 g) for 24 h. After this period, the catheter volumes were removed and the catheters were flushed with 0.9% NaCl, prior to 24 h treatment with either 250 μg/ml fluconazole or saline control. The catheters were then removed from the animals, and biofilms were dislodged by sonication and vortexing. Viable cell counts were determined by dilution plating. In the same animals, kidneys were removed and viable cell burden assessed by dilution plating to estimate dispersion from the vascular catheter. Three animal and culture replicates were used per condition.

### Statistical analysis

Normality of collected data was assessed using Shapiro–Wilk test^42^. Statistically significant outliers were identified based on the Grubbs’ test. Data sets of equal or different sample size were analyzed using non-parametric Kruskal–Wallis one-way analysis of variance with post hoc uncorrected Dunn’s multiple comparison test. When one-way analysis of variance was not feasible, unpaired two-tailed *t*-test was applied. Data were processed with GraphPad Prism 9 for Windows 64-bit (version 9.2.0).

### Ethics statement

All animal procedures were approved by the Institutional Animal Care and Use Committee at the University of Wisconsin-Madison according to the guidelines of the Animal Welfare Act, The Institute of Laboratory Animal Resources Guide for the Care and Use of Laboratory Animals, and Public Health Service Policy. The approved animal protocol number is DA0031. Animals were housed at 22 °C, humidity 45%, and with a 12 h light-dark cycle.

### Reporting summary

Further information on research design is available in the Nature Research Reporting Summary linked to this article.

## Supplementary information

Supplementary Information

Description of Additional Supplementary Files⇐

Supplementary Data 1

Supplementary Data 2

Supplementary Data 3

Supplementary Data 4

Supplementary Data 5

Supplementary Data 6

Supplementary Data 7

Supplementary Data 8

Reporting Summary

## Data Availability

The authors declare that the data supporting the findings of this study are available within the Supplementary Information files. The proteomic data are available via ProteomeXchange with identifier PXD028596. Further requests should be addressed to the corresponding author. Source data are provided with this paper.

## Source data

Source Data

## References

[1] Rodrigues ML (2008). Extracellular vesicles produced by *Cryptococcus neoformans* contain protein components associated with virulence. Eukaryot. Cell.

[2] Deatherage BL, Cookson BT (2012). Membrane vesicle release in bacteria, eukaryotes, and archaea: a conserved yet underappreciated aspect of microbial life. Infect. Immun..

[3] Bielska E (2018). Pathogen-derived extracellular vesicles mediate virulence in the fatal human pathogen *Cryptococcus gattii*. Nat. Commun..

[4] Coelho C (2019). Listeria monocytogenes virulence factors, including listeriolysin O, are secreted in biologically active extracellular vesicles. J. Biol. Chem..

[5] Li Z, Clarke AJ, Beveridge TJ (1998). Gram-negative bacteria produce membrane vesicles which are capable of killing other bacteria. J. Bacteriol..

[6] Lin J (2017). A Pseudomonas T6SS effector recruits PQS-containing outer membrane vesicles for iron acquisition. Nat. Commun..

[7] Prados-Rosales R (2011). Mycobacteria release active membrane vesicles that modulate immune responses in a TLR2-dependent manner in mice. J. Clin. Invest..

[8] Costerton JW, Stewart PS, Greenberg EP (1999). Bacterial biofilms: a common cause of persistent infections. Science.

[9] Pfaller MA, Diekema DJ (2007). Epidemiology of invasive candidiasis: a persistent public health problem. Clin. Microbiol. Rev..

[10] Cleveland AA (2012). Changes in incidence and antifungal drug resistance in candidemia: results from population-based laboratory surveillance in Atlanta and Baltimore, 2008–2011. Clin. Infect. Dis..

[11] Pappas PG (2016). Clinical practice guideline for the management of candidiasis: 2016 update by the Infectious Diseases Society of America. Clin. Infect. Dis..

[12] Mitchell KF, Zarnowski R, Andes DR (2016). Fungal super glue: the biofilm matrix and its composition, assembly, and functions. PLoS Pathog..

[13] Zarnowski R (2018). *Candida albicans* biofilm-induced vesicles confer drug resistance through matrix biogenesis. PLoS Biol..

[14] Juan T, Furthauer M (2018). Biogenesis and function of ESCRT-dependent extracellular vesicles. Semin Cell Dev. Biol..

[15] Schmidt O, Teis D (2012). The ESCRT machinery. Curr. Biol..

[16] Nobile CJ (2012). A recently evolved transcriptional network controls biofilm development in *Candida albicans*. Cell.

[17] Mitchell KF (2015). Community participation in biofilm matrix assembly and function. Proc. Natl Acad. Sci. USA.

[18] Taff HT (2012). A Candida biofilm-induced pathway for matrix glucan delivery: implications for drug resistance. PLoS Pathog..

[19] Andes D (2004). Development and characterization of an in vivo central venous catheter *Candida albicans* biofilm model. Infect. Immun..

[20] Chandra J (2001). Biofilm formation by the fungal pathogen *Candida albicans*: development, architecture, and drug resistance. J. Bacteriol..

[21] Uppuluri P (2010). Dispersion as an important step in the *Candida albicans* biofilm developmental cycle. PLoS Pathog..

[22] Nobile CJ (2008). Complementary adhesin function in *C. albicans* biofilm formation. Curr. Biol..

[23] Ofir-Birin Y, Heidenreich M, Regev-Rudzki N (2017). Pathogen-derived extracellular vesicles coordinate social behaviour and host manipulation. Semin Cell Dev. Biol..

[24] Zarnowski R, Sanchez H, Andes DR (2016). Large-scale production and isolation of *Candida* biofilm extracellular matrix. Nat. Protoc..

[25] Noble SM, Johnson AD (2005). Strains and strategies for large-scale gene deletion studies of the diploid human fungal pathogen *Candida albicans*. Eukaryot. Cell.

[26] Gola S, Martin R, Walther A, Dunkler A, Wendland J (2003). New modules for PCR-based gene targeting in *Candida albicans*: rapid and efficient gene targeting using 100 bp of flanking homology region. Yeast.

[27] Ramage G, Vande Walle K, Wickes BL, Lopez-Ribot JL (2001). Standardized method for in vitro antifungal susceptibility testing of *Candida albicans* biofilms. Antimicrob. Agents Chemother..

[28] Taff HT, Nett JE, Andes DR (2012). Comparative analysis of *Candida* biofilm quantitation assays. Med. Mycol..

[29] Martin SE, Shabanowitz J, Hunt DF, Marto JA (2000). Subfemtomole MS and MS/MS peptide sequence analysis using nano-HPLC micro-ESI Fourier transform ion cyclotron resonance mass spectrometry. Anal. Chem..

[30] Perkins DN, Pappin DJ, Creasy DM, Cottrell JS (1999). Probability-based protein identification by searching sequence databases using mass spectrometry data. Electrophoresis.

[31] Keller A (2002). Experimental protein mixture for validating tandem mass spectral analysis. OMICS.

[32] Babicki S (2016). Heatmapper: web-enabled heat mapping for all. Nucleic Acids Res..

[33] Perez-Riverol Y (2019). The PRIDE database and related tools and resources in 2019: improving support for quantification data. Nucleic Acids Res..

[34] Kumar L, Matthias EF (2007). Mfuzz: a software package for soft clustering of microarray data. Bioinformation.

[35] Glazier VE (2018). Systematic complex haploinsufficiency-based genetic analysis of *Candida albicans* transcription factors: tools and applications to virulence-associated phenotypes. G3.

[36] Nett JE, Cain MT, Crawford K, Andes DR (2011). Optimizing a *Candida* biofilm microtiter plate model for measurement of antifungal susceptibility by tetrazolium salt assay. J. Clin. Microbiol..

[37] Zarnowski R (2014). Novel entries in a fungal biofilm matrix encyclopedia. mBio.

[38] Blakeney AB, Harris PJ, Henry RJ, Stone BA, Norris T (1982). Gas-chromatography of alditol acetates on a high-polarity bonded-phase vitreous-silica column. J. Chromatogr..

[39] Thery C (2018). Minimal information for studies of extracellular vesicles 2018 (MISEV2018): a position statement of the International Society for Extracellular Vesicles and update of the MISEV2014 guidelines. J. Extracell. Vesicles.

[40] Gardiner, C., Ferreira, Y. J., Dragovic, R. A., Redman, C. W. & Sargent, I. L. Extracellular vesicle sizing and enumeration by nanoparticle tracking analysis. *J. Extracell Vesicles* **2**, 10.3402/jev.v2i0.19671 (2013). 10.3402/jev.v2i0.19671 PMC3760643 24009893

[41] Dominguez, E. et al. Conservation and divergence in the *Candida* species biofilm matrix mannan-glucan complex structure, function, and genetic control. *mBio* **9**, 10.1128/mBio.00451-18 (2018). 10.1128/mBio.00451-18 PMC5885036 29615504

[42] Shapiro SS, Wilk MB (1965). An analysis of variance test for normality (complete samples). Biometrika.

