# Supplementary material for: Coordination of fungal biofilm development by extracellular vesicle cargo
Source: Nat Commun. 2021 Oct 29;12:6235. doi: 10.1038/s41467-021-26525-z (PMC8556236; doi:10.1038/s41467-021-26525-z)
Supplement: Supplementary file 1 — Supplementary Information [file 41467_2021_26525_MOESM1_ESM.pdf]

Supplementary Figure 1

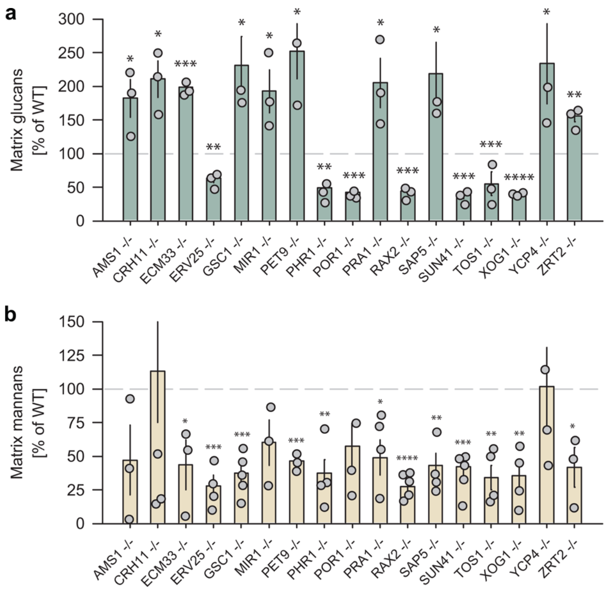

Supplementary Figure 1. Impact of EV protein cargo on biofilm matrix hexose content. a. The percentage changes in biofilm matrix glucans in the tested EV cargo protein mutants determined by gas chromatography. Each dot is an independent biological replicate and represents the mean of 2 technical replicates, from left to right *n* = 3, 3, 3, 3, 3, 3, 3, 5, 3, 3, 3, 3, 3, 3, 3, 3, 3. Error bars represent standard deviation. Non-parametric Kruskal-Wallis one-way analysis of variance with uncorrected Dunn's multiple comparison test was performed. Indicated *p* values, from left to right: 0.0428, 0.0145, 0.0001, 0.0067, 0.0378, 0.0438, 0.0205, 0.0012, 0.0002, 0.0457, 0.0008, 0.0465, 0.0006, 0.0076, <0.0001, 0.0492, and 0.0041. **b.** The percentage changes in biofilm matrix mannans in the tested EV cargo protein mutants determined by gas chromatography. *n*=3-6 replicates. Each dot is an independent biological replicate and represents the mean of 2 technical replicates, from left to right *n* = 3, 6, 3, 4, 5, 3, 3, 5, 3, 4, 5, 4, 5, 4, 4, 4, 3. Error bars represent standard deviation. Non-parametric Kruskal-Wallis one-way analysis of variance with uncorrected Dunn's multiple comparison test was performed. Indicated *p* values, from left to right: 0.0402, 0.0007, 0.0006, 0.0003, 0.0030, 0.0236, <0.0001, 0.0031, 0.0009, 0.0018, 0.0040, and 0.0183.

Supplementary Figure 2

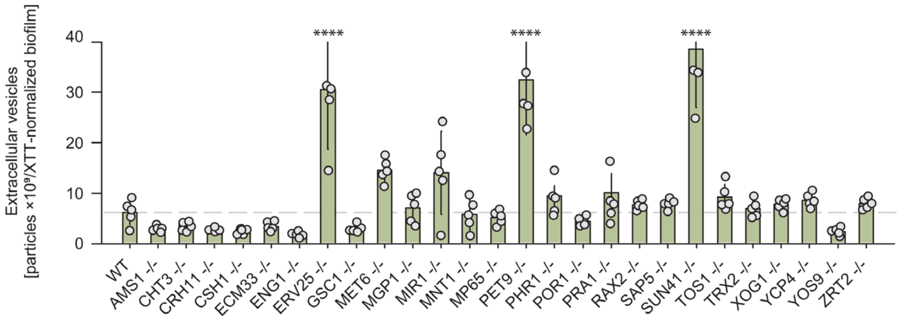

Supplementary Figure 2. Quantitative analysis of biofilm EVs in *Candida* EV cargo mutant biofilms. Concentrations of EVs were measured by imaging flow cytometry and then normalized based on XTT activity of biofilms with reference to WT. Each dot is an independent biological replicate and represents the mean of 5 technical replicates, n = 5. Error bars represent standard deviation. Non-parametric Kruskal-Wallis one-way analysis of variance with uncorrected Dunn's multiple comparison test was performed. Indicated p values are <0.0001.

Supplementary Figure 3

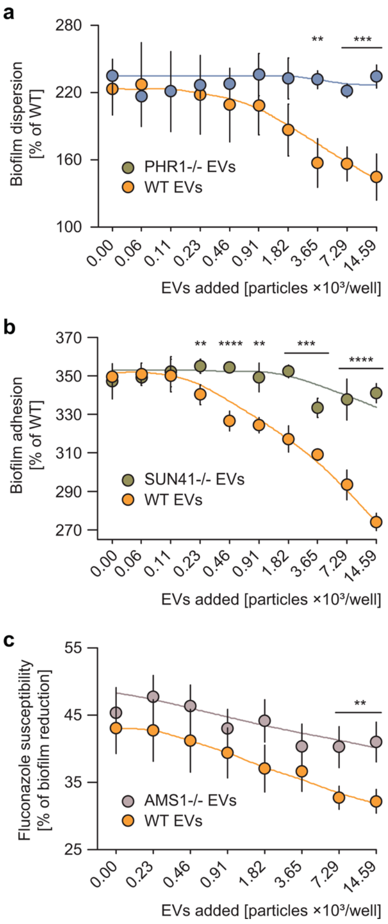

Supplementary Figure 3. Additional WT but not mutant EV reverse cargo mutant biofilm phenotypes. a. Effect of exogenous WT and PHR1 null mutant biofilm EVs on WT and PHR1 null mutant biofilm dispersion as measured by the 96-well XTT assay. Biofilm cultures of select mutant strains with altered phenotypes amended with WT EVs return toward WT capacity, while the mutant EV addition exhibited minimal impact on the biofilm phenotypes. Each dot is an independent biological replicate and represents the mean of 8 technical replicates, $n = 4$. Error bars represent standard deviation. Non-parametric Kruskal-Wallis one-way analysis of variance with uncorrected Dunn's multiple comparison test was performed. Indicated p values, from left to right: 0.0014, 0.0004, and 0.0006. **b.** Effect of exogenous WT and SUN41 null mutant biofilm EVs on WT and SUN41 null mutant biofilm adhesion as measured by the 96-well XTT assay. Biofilm cultures of select mutant strains with altered phenotypes amended with WT EVs return toward WT capacity, while the mutant EV addition exhibited minimal impact on the biofilm phenotypes. Each dot is an independent biological replicate and represents the mean of 8 technical replicates, $n = 4$. Error bars represent standard deviation Non-parametric Kruskal-Wallis one-way analysis of variance with uncorrected Dunn's multiple comparison test was performed. Indicated p values, from left to right: 0.0072, <0.0001, 0.0022, 0.0002, 0.0003, 0.0011, and <0.0001. **c.** Effect of exogenous WT and AMS1 null mutant biofilm EVs on WT and AMS1 null mutant biofilm drug susceptibility as measured by the 96-well XTT assay. Biofilm cultures of select mutant strains with altered phenotypes amended with WT EVs return toward WT capacity, while the mutant EV addition exhibited minimal impact on the biofilm phenotypes. Each dot is an independent biological replicate and represents the mean of 8 technical replicates, $n = 4$. Error bars represent standard deviation. Non-parametric Kruskal-Wallis one-way analysis of variance with uncorrected Dunn's multiple comparison test was performed. Indicated p values are 0.0097 and 0.0043.

Supplementary Figure 4

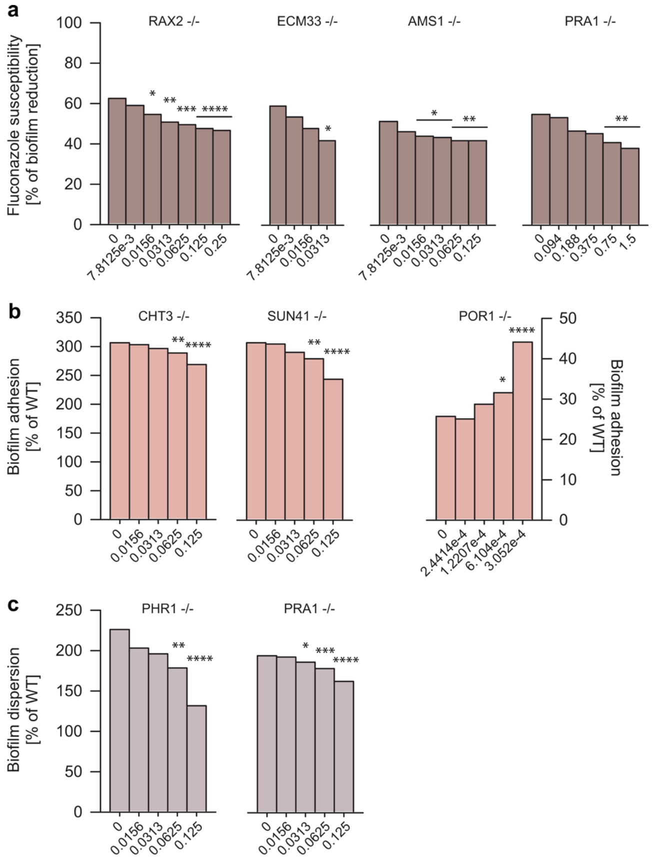

Supplementary Figure 4. Effect of exogenous wild type biofilm EVs on biofilm phenotype. a. Fluconazole susceptibility of the RAX2, ECM33, AMS1, and PRA1 null deletion EV cargo mutants as measured by the 96-well XTT assay. Biofilm cultures of the fluconazole-sensitive mutant strain amended with 2-fold increasing doses of WT type EVs regained their ability to grow in the presence of fluconazole. Similar effects were previously reported for PHR1 and SUN41 mutants. Each dot is an independent biological replicate and represents the mean of 8 technical replicates, n = 4. Error bars represent standard deviation. Non-parametric Kruskal-Wallis one-way analysis of variance with uncorrected Dunn's multiple comparison test was performed. Indicated p values, from left to right: RAX2 : 0.0422, 0.0016, 0.0005, <0.0001, and <0.0001; ECM33: 0.0237; AMS1: 0.0470, 0.0288, 0.0082, and 0.0081; and PRA1: 0.0064 and 0.0011. **b.** Impact of 2-fold increasing concentrations of WT EVs on CHT3, SUN41, and POR1 mutant adhesion in a 96-well XTT assay. The phenotype of both hyper- and hypo-adhesive mutants were returned toward WT in the presence of WT EVs in a dose-dependent manner. Each dot is an independent biological replicate and represents the mean of 8 technical replicates, n = 4. Error bars represent standard deviation. Non-parametric Kruskal-Wallis one-way analysis of variance with uncorrected Dunn's multiple comparison test was performed. Indicated p values, from left to right: CHT3: 0.0020 and <0.0001; SUN41: 0.0051 and <0.0001; POR1: 0.0288 and <0.0001. **c.** Dispersion of the PHR1 and PRA1 null deletion EV cargo mutant as measured by the 96-well XTT assay. Biofilm cultures of these mutants with increased dispersion compared to WT amended with 2-fold increasing doses of WT type EVs returned to WT dispersion levels. Each dot is an independent biological replicate and represents the mean of 8 technical replicates, n = 4. Error bars represent standard deviation. Non-parametric Kruskal-Wallis one-way analysis of variance with uncorrected Dunn's multiple comparison test was performed. Indicated p values, from left to right: PHR1 : 0.0062 and <0.0001; PRA1: 0.0485, 0.0003, and <0.0001.

Supplementary Figure 5

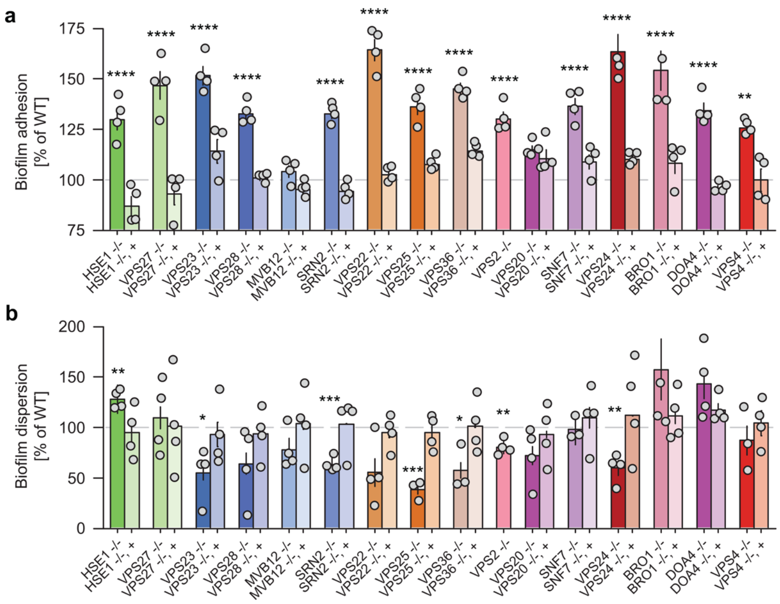

Supplementary Figure 5. ESCRT cargo proteins affect cell adhesion and dispersion from biofilms. a. The percent of biofilm adhesion over 90 min of incubation of the ESCRT mutants as compared to WT control. Both the null deletion mutants and the corresponding complemented strains are shown. Each dot is an independent biological replicate and represents the mean of 8 technical replicates, n = 4. Error bars represent standard deviation. Non-parametric Kruskal-Wallis one-way analysis of variance with uncorrected Dunn's multiple comparison test was performed. Indicated p values, from left to right: <0.0001, <0.0001, <0.0001, <0.0001, <0.0001, <0.0001, <0.0001, <0.0001, <0.0001, <0.0001, <0.0001, <0.0001, <0.0001, and 0.0011. **b.** The percent of fungal cell dispersion from mature biofilms at 24 h. The ESCRT mutants are shown as compared to WT control. Both the null deletion mutants and the corresponding complemented strains are shown. Each dot is an independent biological replicate and represents the mean of 8 technical replicates, from left to right n = 4, 4, 4, 4, 4, 4, 4, 4, 4, 4, 4, 4, 4, 4, 3, 4, 3, 4, 4, 4, 4, 3, 4, 4, 4, 4, 4, 4, 4, 3, 4. Error bars represent standard deviation. Non-parametric Kruskal-Wallis one-way analysis of variance with uncorrected Dunn's multiple comparison test was performed. Indicated p values, from left to right: 0.0034, 0.0328, 0.0004, 0.0004, 0.0311, 0.0074, and 0.0054.

Supplementary Figure 6

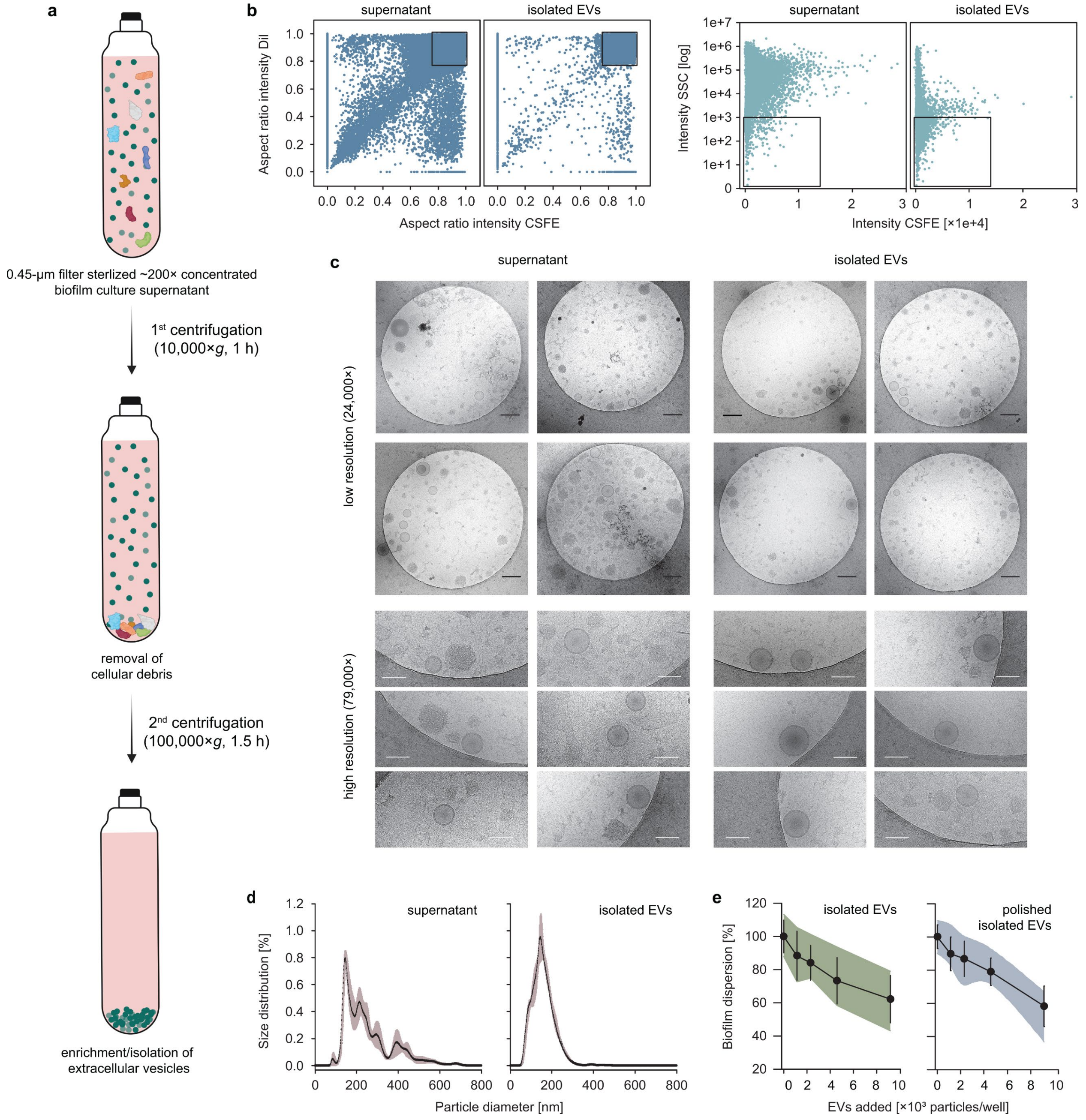

Supplementary Figure 6. Qualitative validation of EV isolation from *Candida* biofilm. a. A schematic of the optimized EV isolation protocol used in this study. Collected supernatant is filter sterilized in order to remove cells, and then concentrated about 200× using a tangential flow system followed by centrifugation at 10,000×*g*, which eliminates most of cellular debris. Collected EV-enriched supernatant is next ultracentrifuged (100,000×*g*), which yields purified EVs. Created with BioRender.com. **b.** Qualitative assessment of concentrated supernatant and isolated EVs by imaging flow cytometry. The CSFE aspect ratio fluorescence intensity (Channel 2) was plotted against the aspect ratio fluorescence intensity (Channel 3) (left hand figures), which demonstrated the distribution of double-strained round-like particles (gated upper square) in the tested samples. Extracellular vesicles (gated lower square) were then selected by plotting the CSFE intensity (Channel 2) against the SSC channel (darkfield/light scattering Channel 6) (right hand figures). EVs were identified as double-stained round-like fluorescent particles,

which are not detectable in the darkfield scattering channel. The results indicate that the applied two-step centrifugation process yields samples enriched in EVs while eliminating most of contaminants. **c.** Cryo-TEM images of concentrated supernatant and isolated EV samples provide visual quality assessment. Low- and high-resolution images visualize the presence of EVs and co-purified contaminants. The latter pool occurs less frequently in the isolated EVs, which validates the EV isolation protocol. Scale bars = 100 nm. **d.** Particle size distribution in concentrated supernatant and isolated EVs fractions as evaluated by dynamic light scattering. Light brown shading represents standard deviation of 5 technical replicates, n = 3. Congruent data were obtained by nanoparticle tracking analysis. **e**. An exemplary of functional validation of purified biofilm EVs. In order to verify whether the observed phenotypic effects in the EV addback assay were driven by the presence of EVs and not due to the occurrence of co-purified non-vesicular contaminants, the isolated EV fraction was further processed using an exoEasy Maxi Kit (Qiagen). This kit uses generic membranes with high affinity for specific binding and recovery of EVs from cell culture supernatant. Such purified EVs were then assessed in the EV addback biofilm assay. Here exogenous EVs were administered to the PHR1 null deletion mutant biofilm at concentrations up to 733,203 ± 46,326 particles/ml and biofilm dispersion was measured. Comparable activity levels of both ultracentrifuged and kit-polished EVs were detected, which indicates that the phenotypic effects are indeed induced by EVs. Thus, we subsequently removed this additional polishing step from our EV purification protocol in this study.Lines represent the mean of 8 technical replicates, n = 3. Dark green and blue gray shaded areas represent minimal and maximal value range distribution. Error bars represent standard deviation. Non-parametric Kruskal-Wallis one-way analysis of variance with uncorrected Dunn’s multiple comparison test was performed. No significant differences were determined.
