# Supplementary material for: Coordination of fungal biofilm development by extracellular vesicle cargo
Source: Nat Commun. 2021 Oct 29;12:6235. doi: 10.1038/s41467-021-26525-z (PMC8556236; doi:10.1038/s41467-021-26525-z)
Supplement: Supplementary file 2 — Description of Additional Supplementary Files⇐ [file 41467_2021_26525_MOESM2_ESM.pdf]

Description of Additional Supplementary Files

File Name: Supplementary Data 1
Description: Exosomal proteomes of C. albicans WT and ESCRT mutants biofilms

File Name: Supplementary Data 2
Description: Comparative proteomics of extracellular vesicles in Candida albicans WT and ESCRT mutants biofilms

File Name: Supplementary Data 3
Description: Candida albicans biofilm EV cargo protein candidates selected using the Mfuzz fuzzy clustering method

File Name: Supplementary Data 4
Description: KEGG/BRITE functional characterization of select Candida albicans biofilm EV cargo protein candidates

File Name: Supplementary Data 5
Description: Candida albicans biofilm EV cargo protein candidates biofilm formation capacity compared to WT reference

File Name: Supplementary Data 6
Description: Planktonic minimum inhibitory concentration for select cargo mutants compared to WT reference

File Name: Supplementary Data 7
Description: Candida albicans strain genotypes used in this study

File Name: Supplementary Data 8
Description: Primer sequences for EV cargo mutant strain construction
