# Supplementary material for: Coordination of fungal biofilm development by extracellular vesicle cargo
Source: Nat Commun. 2021 Oct 29;12:6235. doi: 10.1038/s41467-021-26525-z (PMC8556236; doi:10.1038/s41467-021-26525-z)
Supplement: Supplementary file 11 — Reporting Summary [file 41467_2021_26525_MOESM11_ESM.pdf]

nature portfolio

Corresponding author(s): David Andes and Aaron Mitchell
NCOMMS-21-29673-T

Last updated by author(s): Sep 21, 2021

Reporting Summary

Nature Portfolio wishes to improve the reproducibility of the work that we publish. This form provides structure for consistency and transparency in reporting. For further information on Nature Portfolio policies, see our Editorial Policies and the Editorial Policy Checklist.

Statistics

For all statistical analyses, confirm that the following items are present in the figure legend, table legend, main text, or Methods section.

n/a	Confirmed	
☐	☒	The exact sample size (*n*) for each experimental group/condition, given as a discrete number and unit of measurement
☐	☒	A statement on whether measurements were taken from distinct samples or whether the same sample was measured repeatedly
☐	☒	The statistical test(s) used AND whether they are one- or two-sided<br>*Only common tests should be described solely by name; describe more complex techniques in the Methods section.*
☐	☒	A description of all covariates tested
☐	☒	A description of any assumptions or corrections, such as tests of normality and adjustment for multiple comparisons
☐	☒	A full description of the statistical parameters including central tendency (e.g. means) or other basic estimates (e.g. regression coefficient) AND variation (e.g. standard deviation) or associated estimates of uncertainty (e.g. confidence intervals)
☒	☐	For null hypothesis testing, the test statistic (e.g. *F*, *t*, *r*) with confidence intervals, effect sizes, degrees of freedom and *P* value noted<br>*Give P values as exact values whenever suitable.*
☒	☐	For Bayesian analysis, information on the choice of priors and Markov chain Monte Carlo settings
☒	☐	For hierarchical and complex designs, identification of the appropriate level for tests and full reporting of outcomes
☒	☐	Estimates of effect sizes (e.g. Cohen's *d*, Pearson's *r*), indicating how they were calculated

Our web collection on statistics for biologists contains articles on many of the points above.

Software and code

Policy information about availability of computer code

Data collection	None used for data collection
Data analysis	INSPIRE software from ImageStreamX MkII flow cytometer system was used for vesicle analysis<br>R with Mfuzz package was used for proteomic clustering analysis<br>NanoSight Software NTA 3.4 Build 3.4.003 was used for vesicle analysis<br>GraphPad Prism 9 for Windows 64-ibt version 9.2.0 was used for statistical analysis

For manuscripts utilizing custom algorithms or software that are central to the research but not yet described in published literature, software must be made available to editors and reviewers. We strongly encourage code deposition in a community repository (e.g. GitHub). See the Nature Portfolio guidelines for submitting code & software for further information.

Data

Policy information about availability of data

All manuscripts must include a data availability statement. This statement should provide the following information, where applicable:

- Accession codes, unique identifiers, or web links for publicly available datasets
- A description of any restrictions on data availability
- For clinical datasets or third party data, please ensure that the statement adheres to our policy

The authors declare that the data supporting the findings of this study are available within the Supplementary information files. Source data are provided with this paper. The proteomic data are available via ProteomeXchange with identifier PXD028596. Further requests should be addressed to the corresponding author.

Field-specific reporting

Please select the one below that is the best fit for your research. If you are not sure, read the appropriate sections before making your selection.

☒ Life sciences ☐ Behavioural & social sciences ☐ Ecological, evolutionary & environmental sciences

For a reference copy of the document with all sections, see nature.com/documents/nr-reporting-summary-flat.pdf

Life sciences study design

All studies must disclose on these points even when the disclosure is negative.

Sample size	An initial sample size of 3 was chosen for plate assays to allow determination of mean and standard error for each assay. A sample size of 3 was chosen for in vivo studies as a clinical proof of principle and sample size limited by available resources.
Data exclusions	No data were excluded
Replication	Biological replicates were repeated at least 3 times and all resplicates are reported
Randomization	We did not perform randomization as we were not comparing therapies.
Blinding	Blinding was not performed as it is unclear what we would blind.

Reporting for specific materials, systems and methods

We require information from authors about some types of materials, experimental systems and methods used in many studies. Here, indicate whether each material, system or method listed is relevant to your study. If you are not sure if a list item applies to your research, read the appropriate section before selecting a response.

Materials & experimental systems

n/a	Involved in the study	
☒	☐	Antibodies
☒	☐	Eukaryotic cell lines
☒	☐	Palaeontology and archaeology
☐	☒	Animals and other organisms
☒	☐	Human research participants
☒	☐	Clinical data
☒	☐	Dual use research of concern

Methods

n/a	Involved in the study	
☒	☐	ChIP-seq
☒	☐	Flow cytometry
☒	☐	MRI-based neuroimaging

Animals and other organisms

Policy information about studies involving animals; ARRIVE guidelines recommended for reporting animal research

Laboratory animals	Sprague Dawley rats, 16 week, 400 g, femal
Wild animals	Study did not involve wild caught animals
Field-collected samples	The studies did not involve field collected samples.
Ethics oversight	All animal procedures were approved by the Institutional Animal Care and Use Committee at the University of Wisconsin-Madison according to the guidelines of the Animal Welfare Act, The Institute of Laboratory Animal Resources Guide for the Care and Use of Laboratory Animals, and Public Health Service Policy. The approved animal protocol number is DA0031.

Note that full information on the approval of the study protocol must also be provided in the manuscript.
